# The Effect of Teacher Autonomy Support on Leisure-Time Physical Activity via Cognitive Appraisals and Achievement Emotions: A Mediation Analysis Based on the Control-Value Theory

**DOI:** 10.3390/ijerph18083987

**Published:** 2021-04-10

**Authors:** Julia Zimmermann, Henri Tilga, Joachim Bachner, Yolanda Demetriou

**Affiliations:** 1 Professorship of Educational Science in Sport and Health, Department of Sport and Health Sciences, Technical University of Munich, 80992 Munich, Germany; joachim.bachner@tum.de (J.B.); yolanda.demetriou@tum.de (Y.D.); 2 Institute of Sport Science and Physiotherapy, Faculty of Medicine, University of Tartu, 50411 Tartu, Estonia; henri.tilga@ut.ee

**Keywords:** achievement emotions, control-value theory, autonomy support, self-efficacy, intrinsic value, enjoyment, anxiety, physical activity, physical education

## Abstract

Analyzing students’ emotional experience in physical education (PE) is of crucial importance as it may fill an important gap in research examining the role of PE for students’ leisure-time physical activity (PA). Based on the control-value theory of achievement emotions, the purpose of this study was to test the assumption that multi-dimensional autonomy support of the PE teacher may affect students’ leisure-time PA via their appraisals of control and value and achievement emotions experienced in PE. Variance-based structural equation modelling was used to test the proposed model in a sample of 1030 students aged between 11 and 18 years (M = 13.4, SD = 1.48) stemming from schools with the lowest educational level among secondary schools in Germany. The results indicated that in particular cognitive autonomy support positively predicted students’ self-efficacy and intrinsic value. Whereas appraisals of self-efficacy were negatively related to the experience of anxiety, intrinsic value was a major positive predictor of enjoyment. Enjoyment, in turn, was of substantial relevance for leisure-time PA. The findings offer a meaningful contribution in understanding students’ emotional experiences and remind PE teachers of their opportunity to adopt an autonomy-supportive teaching style to positively influence the emotions of their students.

## 1. Introduction

The health benefits of regular physical activity (PA) for children and adolescents, such as lower risk of being overweight or obesity, type II diabetes mellitus and metabolic syndrome, are widely known [1,2]. Additionally, the findings suggest that high levels of PA and low levels of sedentary behavior are related to better mental health in children and adolescents [3,4]. However, self-report studies have shown that only 19% of students worldwide aged 11 to 17 fulfil the World Health Organization (WHO) recommendation of a daily average of 60 min of moderate-to-vigorous PA per day across the week [5,6]. Results of the German Health Interview and Examination Survey for Children and Adolescents (KiGGS) indicated that in Germany only 22.4% of girls and 29.4% of boys aged 3 to 17 reach the WHO guideline and that PA decreases significantly from age 3 to 17 [7]. Furthermore, PA over the life course is subject to a tracking effect, meaning that PA during adolescence is positively associated with PA in adulthood [8].

These findings suggest that promotion of PA in adolescents should be a priority for policymakers, parents and teachers [9]. This in turn points to the relevance of physical education (PE). PE exhibits great potential in fostering a healthy lifestyle since students take part in PE lessons regardless of their PA level, personal attitudes, previous experiences or socioeconomic background. Therefore, PE should be used as a platform for increasing students’ commitment and decreasing their dropout rates in physical activities and sports in a lifelong perspective. This refers particularly to students of low socioeconomic background since socioeconomic status (SES) has been found to be positively related to leisure-time PA [10]. Furthermore, there are indications that children and adolescents from families with low SES are significantly more likely to have a moderate, bad or very bad health status than their peers from families with high SES [11].

However, based on ambiguous findings, there is substantial concern if and to what extent PE is able to positively affect students’ PA during leisure time [12,13]. In line with the overall finding that intervention studies focusing on PA behavior change often failed to increase PA [14,15], intervention programs specifically implemented in PE mostly reached small effects on leisure-time PA [16,17,18]. This may partly be due to the theories that are mainly used in intervention design, most of them being of social-cognitive or humanistic nature [14,19]. While the applied theories still vary to some extent, they share the common neglect of affect and subjective emotional experiences students make during PE lessons. Intrinsic motivation, one of the central constructs of the often used self-determination theory (SDT; [20]), does relate closely to the concept of emotions [21]. However, although the extent of intrinsic motivation and the experience of emotions can be explained by similar needs and triggers [22], they still are conceptually different. Whereas motivation refers to the energy that drives a specific action, emotions describe the physiological and psychological processes determining the subjective experiences while engaging in a certain behavior [21].

Over the last years, research has focused more and more on affect-related concepts to understand and promote PA behavior change [23,24,25] and evidence for the relation between affective experiences and PA already exists [15,26]. Emotions, affective associations and ratings of pleasure and displeasure during exercise have been identified as significant correlates and predictors of sport and exercise behavior [27,28]. Nevertheless, until recently, the number of interventions explicitly focusing on emotions and affective experiences to increase exercise maintenance or long-term PA behavior was small [14,29]. To summarize, by considering students’ subjective emotional experiences, a more holistic understanding of the processes taking place in PE lessons could be attained. Consequently, if theory-guided PE interventions want to successfully change the experience of PA and promote fundamental motivation for PA, the role of affect, feelings and emotions experienced in PE must form part of the theories underlying these interventions as well [30,31,32]. Therefore, alternative theoretical models need to be taken into account [25,33].

Complementary to social-cognitive or humanistic theories, Brand and Ekkekakis [34] have introduced an alternative model with a dual-process theory that concentrates on the psychological processes that guide behavior and focuses specifically on exercise-related feelings. The affective-reflective theory of physical inactivity and exercise (ART) wants to explain and predict behavior in situations where people either remain in a state of physical inactivity or initiate an action. People tend to repeat behavior if they experience joy, while on the other hand, negative emotional experiences decrease the probability of repeated, and thus regular PA [28,34]. Applied to the PE context, it can be assumed that automatic affective evaluations and remembered emotions regarding PA and sports are partially influenced by experiences made in PE and that these experiences may thus have decisive effects on lifelong activity behavior. Therefore, positive and negative experiences in PE can be seen as highly relevant for long-term activity behavior [35]. PE teachers should aim to facilitate the experience of positive emotions while reducing the frequency of negative affect.

To be able to generate emotional experiences that may eventually trigger regular PA in leisure time, an overview of emotions that are potentially experienced by students during PE as well as their determinants and consequences is needed. The control-value theory of learning and achievement emotions (CVT; [36]) serves as an appropriate and established theoretical framework as it presents antecedents and outcomes of emotions in school settings. The CVT comprises a range of distinct achievement emotions, which specifically occur within achievement settings like school [37]. Pekrun [36] describes and classifies achievement emotions according to three major dimensions. The first dimension differentiates between positive and negative valence of an emotion. Whether the emotion is of activating or deactivating character is described by means of the second dimension. The third dimension specifies the object focus and indicates whether the emotion refers to an achievement activity (e.g., learning) or an achievement outcome, namely success or failure. The achievement emotions represented in the CVT reflect both positive and negative affect. Anxiety, anger, shame and boredom are examples for achievement emotions of negative affect. Enjoyment and pride are examples for positive affect. With regard to the three major dimensions proclaimed by Pekrun [36], these emotions can be classified more specifically. Enjoyment while studying, for example, is thus classified as being of positive valence, activating character and related to an achievement activity. Anxiety is classified as a negative, activating and outcome-related achievement emotion.

In previous studies, enjoyment has often been used as an indicator for general positive affect, comprising feelings of fun and pleasure [38,39]. However, it is important to highlight the difference between distinct emotions and global affect. Emotions are specifically related to a given task and have different antecedents [40]. Additionally, disentangling distinct emotions offers a higher precision in the description of students’ emotional experiences compared to the report of general positive and negative affect [41,42]. Furthermore, the predictive power of distinct achievement emotions is higher than the one of global tendencies in affect [43]. Therefore, despite minor conceptual similarities between the achievement emotions, they should be considered as discrete, separate manifestations of emotion.

According to the CVT, the most proximal antecedents of students’ emotional experience in achievement situations are students’ subjective appraisals regarding control and value. These cognitive appraisals are in turn influenced by the specific characteristics of the learning environment [44]. Thus, students’ control and value appraisals are seen as the constructs mediating the link between the characteristics of the learning environment and the experience of distinct achievement emotions [44].

Control-related appraisals refer to students’ competence beliefs, attributional style and their expectancies. According to Pekrun [36], three types of expectancies can be differentiated. Situation-outcome expectancies and action-outcome expectancies refer to the general controllability of a situation and possible effects of an action. Action-control expectancies are relevant one step before, when students appraise whether they are able to initiate and perform an action. Self-efficacy expectation, as it is introduced by Bandura [45], is highly similar to the concept of action-control expectancies and is most popular in representing control appraisals [36]. Value appraisals represent the perceived value of an achievement. These value appraisals can be seen with regard to intrinsic aspects, when the achievement is rated in terms of internal reasons, such as the personal interest attached to it. On the other hand, extrinsic value reflects the relevance of an achievement because of external reasons like a desirable reward [46]. Students’ appraisals of control and value as well as the interaction of the two appraisal dimensions are assumed to determine which emotions are experienced and to which extent. Generally, positive appraisals of control and value regarding a given achievement activity are expected to provoke positive activity emotions, such as enjoyment of studying, and decrease negative activity emotions like anger [36]. However, high scores on value appraisals regarding an achievement outcome, for instance failure in an exam, can also lead to negative outcome emotions like shame or anxiety when paired with negative appraisals of control [36,47]. Several studies have supported the role of control and value appraisals as predictors of achievement emotions [48,49]. In a sample of high school students, control and value appraisals in PE were positive predictors of enjoyment and negative predictors of boredom [50].

Although control-value appraisals reflect personal convictions, they are not unchangeable. In fact, the CVT proposes that antecedents of cognitive appraisals can be identified in the learning environment. Pekrun et al. [48] draw a theoretical link between the SDT with its basic psychological needs [20] and the control-value appraisals [36]. It is assumed that autonomy support influences the cognitive appraisals [36]. Thus, autonomy support provided by the teacher may represent one important aspect of the learning environment. Generally, the way teachers structure the learning environment can form students’ beliefs regarding class-related experiences [51]. It is assumed that if teachers manage to empower their students to take important learning decisions by themselves, their cognitive appraisals should be enhanced [21,36]. Furthermore, self-controlled actions are suggested to facilitate the development of convictions of internal control [44]. Despite conceptual relations between autonomy support and subjective determinants of emotions, the association of autonomy support by the teacher and students’ appraisals of control and value has not been empirically examined in the context of school PE [52]. However, the effects of autonomy support on constructs of control and value have been examined in other educational settings [53,54,55]. Findings indicated that autonomy-supportive teaching enhances students’ ratings of self-efficacy and intrinsic value.

The examination of achievement emotions and their antecedents in PE is no end in itself but may imply insights into highly relevant consequences, such as performance- and health-related outcomes. The CVT assumes emotions to be crucial for understanding student motivation [44]. It is further proclaimed that the achievement emotions students experience in educational settings influence achievement outcomes, such as their performance [48]. Engagement in regular PA can be seen as a performance-related achievement outcome of PE. In line with the theoretical assumptions of the CVT, studies conducted in the context of PE indicate that the experience of enjoyment in PE is related with PA engagement both in PE [56,57] and during leisure time [56,58,59]. With regard to the age effect underlying the development of PA behavior in childhood and adolescence [5,60], enjoyment has been found to delay or even prevent the decline of motivation for PA [61]. Considering emotions of negative affect, emotional experiences may also contribute to a decrease in PA. For example, anxiety has been shown to be related to negative thoughts about PA engagement and consequentially was negatively associated to PA [61,62]. Furthermore, anxiety has been identified as a barrier to future PA engagement [63]. Whereas the experience of anger was not related to PA, anxiety and boredom as a joint representation of negative affect was negatively related to PA, yet without further insights into the separate predictive contributions of anxiety and boredom [64].

Besides direct effects, the CVT also proposes indirect effects between its variables, with the effect of the learning environment on student emotions mediated by control-value appraisals being the most important one [36]. Empirical support for the proposed mediation effect with teacher autonomy support representing the learning environment was found in the context of sports. In a sample of university students attending tennis courses, control and value appraisals mediated the positive indirect effect of teacher autonomy support on enjoyment as well as the negative indirect effect on boredom [52]. In a sample of middle school students, the CVT-based mediation effect could also be supported. Students’ self-efficacy in math and the intrinsic value they assigned to the subject mediated the relationship between teacher autonomy support and boredom [54]. Additionally, since the CVT further assumes that mediated effects of the learning environment do not necessarily end with achievement emotions [36,40], Wang et al. [54] also included academic engagement as an achievement outcome in their analysis. They could show that teacher autonomy support indirectly affected students’ academic engagement in math via self-efficacy, intrinsic value and boredom.

While there are approaches that aim to identify facilitators of emotions to explain exercise maintenance [22,65], so far, the role of emotions has rarely been examined in PA settings [40,61]. Furthermore, key factors that lead to a positive emotional response in a sporting environment are still far from being fully understood [22,33] and there are few empirical findings how potential key factors may be manipulated [14,65]. In order to examine the widely unknown influence of students’ emotional experience in PE on leisure-time PA behavior, distinct achievement emotions have to be measured in PE-specific manner [40] and potential ways to evoke PA-enhancing achievement emotions have to be examined.

**Figure 1:**
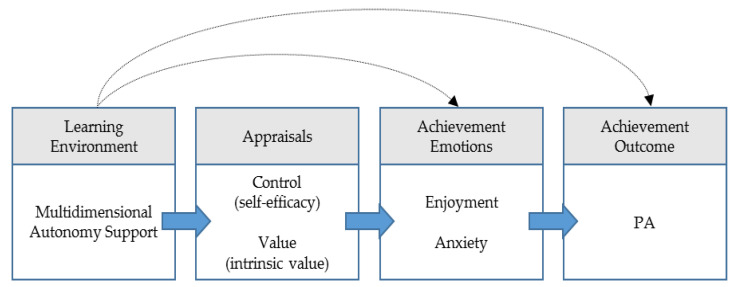
Hypothesized model of the present study based on the control-value theory (CVT) (Pekrun, 2006).

Therefore, using the CVT as a theoretical framework, we want to examine how the learning environment in PE predicts student leisure-time PA via appraisals of control and value and achievement emotions. This is examined in a high-risk sample for physical inactivity comprising lower-track secondary school students mainly stemming from households with a low SES. With regard to previously scarce insights in the specific PE context, teacher autonomy support will represent the learning environment. Self-efficacy and intrinsic value will reflect students’ appraisals of control and value, respectively. Enjoyment and anxiety have been chosen as distinct emotions, since they frequently emerge in achievement settings. Furthermore, by means of this selection both activity- and outcome-related emotions of positive and negative affect are represented [36] (Figure 1). We hypothesize that students perceived autonomy support by the PE teacher is positively related to their appraisals of self-efficacy and intrinsic value (Hypothesis 1). Further, we hypothesize that self-efficacy and intrinsic value exhibit positive associations with enjoyment and negative associations with anxiety (Hypothesis 2). Subsequently, it is hypothesized that enjoyment relates positively while anxiety relates negatively to students’ leisure-time PA (Hypothesis 3). Finally, we hypothesize that perceived teacher autonomy support exhibits a positive indirect relationship with leisure-time PA that is mediated by students’ control-value appraisals and their experience of achievement emotions in PE (Hypothesis 4).

## 2. Materials and Methods

### 2.1. Participants

The study sample comprised 1030 students aged between 11 and 18 years (M = 13.4, SD = 1.48), 408 participants were female (39.6%), 622 participants were male (60.4%). The students attended grades 6 through 10 of the German Mittelschule, which is the school form with the lowest educational level among secondary schools in Germany. The participants stemmed from three urban, three semi-rural and four rural schools. For 51.8% of the participants, German was the language spoken with family members at home. Predominant use of a foreign language at home was indicated by 26.7% of the students. The remaining 20.9% of the participants spoke German and another language to similar extents at home. The average value for SES was at 41.3 (SD = 12.8, n = 991). Thus, SES was substantially lower than in large-scale studies with German-speaking samples, such as the Programme for International Student Assessment (PISA) study, which indicated a mean SES of 51.8 (SD = 21.0, n = 4346) for its participants of grades 7 through 10. Age- and sex-dependent BMI percentiles were used to define cut-off points. Mean percentile (%) was 79 and 86 for girls and boys, respectively, which is in the range of normal weight [66]. All students participated in mandatory single-sex PE lessons for two school hours per week.

### 2.2. Measures

#### 2.2.1. Pilot Study

The questionnaire used in this study was thoroughly pilot tested in advance. In the pilot study, 193 students (11 classes) of grades 6 through 10 from one urban and one rural German Mittelschule completed the questionnaire. By means of the pilot study, the general feasibility of a questionnaire study in a sample of academic underachievers mainly stemming from households of low SES was tested. Furthermore, the pilot study was conducted to gather insights regarding the applicability of the translated and adapted items and the response format. To obtain these insights, participants of the pilot study were to give a signal to the members of the assessment team when they experienced difficulties in understanding or responding to items. After completion of the questionnaire, two academically over-performing and two academically under-performing students of each class participated in structured cognitive interviews [67]. The interviews were conducted with each student separately and took place outside of the classroom, so that the students could express their opinion freely and independently from their classmates. In response to the insights of the pilot study, the wording of some items was slightly adapted. Another important output of the pilot study was a manual that was designed to help the assessment team of the main study to answer consistently to possible questions of the participants about the items and the procedure.

#### 2.2.2. Autonomy Support by PE Teacher

Students rated the perceived autonomy support provided by the PE teacher on the German Multi-Dimensional Perceived Autonomy Support Scale for Physical Education (MD-PASS-PE; [68]). Based on the assumption that there are multiple ways for teachers to support student autonomy [69], the MD-PASS-PE comprises the three subscales cognitive, procedural and organizational autonomy support with each of them containing five items [70]. Cognitive autonomy support refers to the promotion of students’ responsibility for their own learning process. An English example item is “My PE teacher is interested in what students want to do.” Procedural autonomy support is defined as the promotion of students’ participation in deciding how the teaching and learning process is conducted. An example item is “My PE teacher explains why we learn certain exercises.” Organizational autonomy support represents the promotion of students’ responsibility to manage their learning environment. An example item is “My PE teacher allows me to choose sport equipment.” Participants rated the items on a 7-point Likert scale from 1 = strongly disagree to 7 = strongly agree. Items were translated by means of the back-translation technique [71]. Thereby, the original items of the English version were translated into German by a team of bilingual native speakers and experts from the field of sports pedagogy. The translated items were then back-translated into English by another team of bilingual native speakers. Finally, this version was compared with the original English items. A difference in one item was solved by a committee of bilingual researchers.

The German MD-PASS-PE represents a reliable measurement instrument with Cronbach’s alpha values of the three subscales ranging between 0.72 and 0.81 in the validation study [68]. Evidence for factorial validity was given since the assumed three-factor structure was supported within a bi-factor model comprising three specific group factors next to a general factor [68,70,72].

#### 2.2.3. Academic Self-Efficacy in PE

To measure self-efficacy in PE, a German 5-item scale originally developed to measure general academic self-efficacy was used [73]. The items were adapted to the context of PE. Participants responded by means of a 4-point Likert scale. An example item in English would be “If I am asked to perform something challenging in PE class, I believe I will be able to do it.”

#### 2.2.4. Intrinsic Value of PE

The intrinsic value that students ascribe to PE was measured by means of a German 6-item scale. The original scale measured the intrinsic value of mathematics, providing good internal consistency and acceptable factorial validity [74]. Therefore, items were adapted to the PE context. Students responded by use of a 5-point Likert scale. An example item in English would be “No matter what grades I get, PE is very important to me.”

#### 2.2.5. Achievement Emotions in PE

The achievement emotions enjoyment and anxiety were assessed with five items, respectively, which were taken from the Achievement Emotions Questionnaire (AEQ; [75]) and the Achievement Emotions Questionnaire—Mathematics (AEQ-M; [76]). The AEQ provides items for the assessment of achievement emotions as a trait in three different academic achievement settings, i.e., during class, while studying and in exams. In this study, items assessing achievement emotions during class were used. The items were adapted to the context of PE and slightly simplified in terms of the used vocabulary. Students responded on a 5-point Likert scale. An English example item for enjoyment is “I enjoy being in class.” An example item for anxiety is “Thinking about class makes me feel uneasy.” Internal consistency scores of the enjoyment and anxiety subscales in the original AEQ were 0.85 and 0.86, respectively. Factorial validity of the subscales was supported by means of structural equation modeling [75].

#### 2.2.6. Physical Activity

Leisure-time PA of the participants was assessed by means of the 6-item physical activity subscale of the German Physical Self-Description Questionnaire (PSDQ; [77]). Internal consistency of the subscale was tested with three different samples. Cronbach’s alpha values ranged between 0.90 and 0.95. Factorial validity of the PSDQ was supported by means of confirmatory factor analyses [77].

#### 2.2.7. Socioeconomic Status

In order to estimate the socioeconomic status, the students had to indicate their parents’ current jobs and had to provide a short description of the jobs. The classification of the responses was conducted with regard to the International Socioeconomic Index of Occupational Status (ISEI), which is based on the International Standard Classification of Occupation 2008 (ISCO-08) [78]. If an ISEI value could be assigned to the occupations of both parents, the higher value was considered. ISEI values range on a scale from 10 to 89 with higher values indicating a higher SES. Not every participant could be assigned an ISEI value since some students did not know or could not clearly describe their parents’ jobs.

### 2.3. Procedures 

The study was conducted in accordance with the Declaration of Helsinki and was approved by the ethics commission of the Technical University of Munich (304/19 S) and the supervisory school authorities in charge. After receiving these approvals, school principals of the participating school district were provided with study information documents for teachers, parents and students. Afterwards, interested schools were sent consent forms several weeks before the scheduled beginning of the data assessments. Students did only participate if they themselves, their parents, their PE teacher and the school principal had provided positive consent forms. Neither students nor schools were rewarded for study participation in any form. Students could leave out questions if they did not want to answer and they could withdraw their participation at any time before, during or after data collection without any consequences.

The paper-and-pencil data collection was conducted during regular school lessons. Students took on average 35 min to complete the questionnaire. Data assessments did not take place directly after PE lessons to make sure that the assessed PE-related constructs represented trait measures instead of state measures. Before participants started to complete the questionnaire, the head of the assessment team and a research assistant informed the participants about the purpose and the procedure of the assessment. Using example items whose content was independent of the assessment subject, students were also explained how to handle the different response scales. The participants were explicitly informed about the fact that the assessments were not about whether they personally like their teacher or appreciate the general teaching style. Instead, the participants were asked to indicate their approval to each statement separately with regard to the specific content and context of the items. Short paragraphs were included in the questionnaire to help the participants in setting the focus on the respective content and context of the different scales. During completion of the questionnaire, students could give a signal at any time and quietly ask questions in case of problems in understanding the items. After a student had completed the questionnaire, he/she went outside of the classroom where two other research assistants measured height and weight with a stadiometer and a digital weight scale. The described procedures applied to the preparation and implementation of both pilot and main study.

### 2.4. Statistical Analysis

Variance-based structural equation modeling (VB-SEM), also known as partial least squares analysis, was used to test the proposed model by using the Warp PLS v7.0 software [79]. The advantage of VB-SEM is that it is distribution-free and less affected by non-normality, model complexity and smaller sample sizes because it is based on ranked rather than ordinal data [80]. Arithmetic mean imputation was used to handle missing data.

Discriminant validity of the latent variables is considered as given if the square root of the average variance extracted (AVE) for each latent variable exceeds its correlation coefficient with the other latent variables. The overall model fit was assessed using multiple criteria: the goodness-of-fit (GoF) index with values of 0.100, 0.250, and 0.360, corresponding to small, medium, and large effect sizes, respectively [81], the average variance inflation factor (AVIF) value for model parameters, which should be below 5.000 [82], and average R^2^ (ARS) and average path coefficient (APC), which are both expected to be significantly different from zero for an adequate model. Hypothesized mediation effects were tested by calculating indirect effects using a “Table 3” method to increase accuracy and statistical power as suggested by Kock [83]. The dataset analyzed for this study is provided as Supplementary File S1.

## 3. Results

### 3.1. Preliminary Analysis

Skewness (range = −0.918 to 1.575) and kurtosis (range = −0.816 to 2.616) values of all latent variables were within the acceptable range [84], which supported the assumption of normality of the variables included in this analysis. Correlations between the latent variables and composite reliability coefficients are presented in Table 1. All the composite reliability coefficients were on the acceptable level. Discriminant validity was given for every variable. GoF statistics demonstrated a very good overall fit of the proposed model with the data according to fit indices. The GoF index was at 0.400. The AVIF value for the model parameters was 1.642. ARS and APC were at 0.266 and 0.218, respectively (both *p* < 0.001). Factor loadings of the items on the latent variables were at least 0.56, no substantial cross-loadings were identified.

### 3.2. Main Analyses

#### 3.2.1. Direct Effects

**Figure 2:**
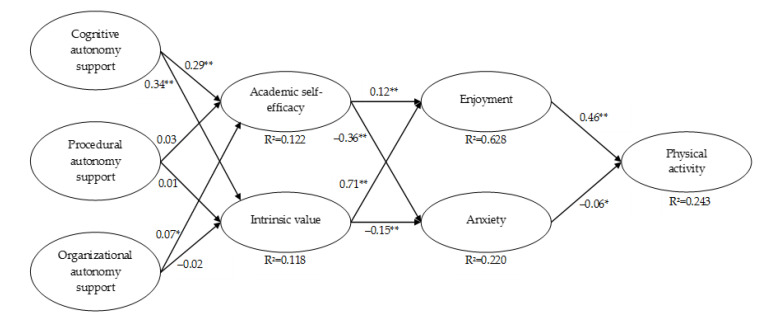
Standardized path coefficients for the variance-based structural equation model. * = *p* < 0.05, ** = *p* < 0.001.

Direct effects of the proposed model are presented in Figure 2. Statistically significant effects are described in the following. Perceived cognitive autonomy support provided by the PE teacher was a positive predictor of students’ academic self-efficacy in PE (β = 0.29, *p* < 0.001, R^2^ = 0.10) and of the intrinsic value students ascribe to PE (β = 0.34, *p* < 0.001, R^2^ = 0.12). Perceived organizational autonomy support provided by the PE teacher also positively predicted students’ academic self-efficacy in PE (β = 0.07, *p* = 0.01, R^2^ = 0.02). Thus, Hypothesis 1 was supported, particularly with respect to cognitive autonomy support as a predictor of control and value appraisals.

While students’ academic self-efficacy in PE positively predicted their enjoyment in PE (β = 0.12, *p* < 0.001, R^2^ = 0.07), it had a negative effect on their anxiety in PE (β = −0.36, *p* < 0.001, R^2^ = 0.16). The intrinsic value that students ascribe to PE also positively predicted students’ enjoyment (β = 0.71, *p* < 0.001, R^2^ = 0.56) and negatively predicted their anxiety in PE (β = −0.15, *p* < 0.001, R^2^ = 0.06). Therefore, Hypothesis 2 was supported.

Finally, students’ enjoyment in PE was a positive predictor of their PA during leisure time (β = 0.46, *p* < 0.001, R^2^ = 0.23). Students’ anxiety in PE negatively predicted their PA level in leisure time (β = −0.06, *p* = 0.02, R^2^ = 0.02). Thus, Hypothesis 3 was supported as well.

#### 3.2.2. Indirect Effects

Statistically significant indirect effects and the respective effect sizes are presented in Table 2. The effects are briefly described in the following. Perceived cognitive autonomy support provided by the PE teacher positively predicted students’ enjoyment in PE via students’ academic self-efficacy in PE and the intrinsic value they ascribe to PE (β = 0.27, *p* < 0.001). Moreover, perceived cognitive autonomy support negatively predicted students’ anxiety in PE via their PE-related academic self-efficacy and the intrinsic value of PE (β = −0.15, *p* < 0.001).

Enjoyment and anxiety in PE mediated the positive effect of students’ academic self-efficacy in PE on their leisure-time PA (β = 0.08, *p* = 0.006). Likewise, enjoyment and anxiety also mediated the positive effect of the intrinsic value students ascribe to PE on PA in leisure time (β = 0.34, *p* < 0.001).

Finally, perceived cognitive autonomy support provided by the PE teacher positively predicted students’ PA in leisure time via students’ PE-related academic self-efficacy, intrinsic value, enjoyment and anxiety (β = 0.14, *p* < 0.001). Thus, Hypothesis 4 was supported.

## 4. Discussion

### 4.1. General Discussion

This study analyzed the relationship of multidimensional teacher autonomy support in PE and leisure-time PA mediated by the PE-related cognitive appraisals academic self-efficacy and intrinsic value and the achievement emotions enjoyment and anxiety. Using VB-SEM in the data of students from grades 6 through 10 of German lower-track secondary schools, the proposed chain of effects was supported. Autonomy support provided by the PE teacher was a positive predictor of PE-related cognitive appraisals, explaining 12.2% and 11.8% of the variance in self-efficacy and intrinsic value that students associate with PE, respectively. The control-value appraisals in turn acted as significant predictors of PE-related achievement emotions, together explaining 62.8% of enjoyment and 22% of anxiety. Finally, achievement emotions experienced in PE significantly predicted students’ PA during leisure time, with 24.3% of the variance in PA being explained by the emotions. Besides supporting the hypothesized direct effects, results also indicated several indirect effects. Cognitive autonomy support exhibited an indirect effect on achievement emotions via cognitive appraisals as well as on leisure-time PA via cognitive appraisals and achievement emotions. Furthermore, the appraisals had an indirect effect on leisure-time PA via achievement emotions in PE.

As assumed in Hypothesis 1, students’ perceived autonomy support provided by the PE teacher was positively related to their appraisals of self-efficacy and intrinsic value. This finding is not only in line with theoretical assumptions [36,45], but also with the findings of previous empirical studies which showed that autonomy support positively predicted appraisals of control and value in other academic contexts [54,55]. These findings suggest that if students are provided the opportunity to influence their learning environment, they tend to have higher action-control expectancies and assign more relevance to the subject of PE. These relations can be corroborated with regard to conceptual considerations about autonomy support, self-efficacy and intrinsic value. With regard to teacher strategies, autonomy-supportive teachers convey an interpersonal message of support and try to understand and adopt the students’ perspective [85]. They provide students with choice, make them feel understood and allow criticism. Available strategies for teachers aiming to promote students’ self-efficacy are similar in that they comprise an honest and open communication, the provision of constructive feedback and the intention to motivate the students to try their best [45,86]. Due to these similarities in the teacher strategies to promote autonomy and self-efficacy, it is likely that successful autonomy support leads to higher appraisals of self-efficacy. The relation between autonomy support and intrinsic value might mainly be attributed to the concept of interest that is shared by the two constructs. Teachers who successfully support students’ autonomy develop student resources that are necessary for their motivation, such as their interest [87,88]. Interest, in turn, is considered a main reason for students to assign a high intrinsic value to an activity or achievement [36,75]. Thus, autonomy support may inherently have a positive effect on the intrinsic value of the learning activity.

The studies that have previously examined the relationship of autonomy support and appraisals of control and value in other academic contexts [54,55,89] measured autonomy support in a unidimensional way, which equals the assessment of cognitive autonomy support [68,70]. In the present study, autonomy support was assessed in a multidimensional way. The results indicate a major role of cognitive autonomy support, which was a significant predictor of both self-efficacy and intrinsic value. While organizational autonomy support was still a significant predictor of self-efficacy, procedural autonomy support was not a relevant factor in the present model. Organizational autonomy support includes aspects like developing rules together, or the choice of group members, equipment and exercise place [69,70]. Being responsible for managing their learning environment, students are supported in making their own decisions, which might make them feel more in control and, more specifically, more self-effective [86]. Contrarily, students’ involvement in how the learning process is arranged, which was assessed through procedural autonomy support [69] does not strengthen the selected control and value appraisals.

The control and value appraisals served as proximal antecedents of discrete achievement emotions, which is in line with the CVT [36]. In accordance with Hypothesis 2, self-efficacy and intrinsic value showed positive relations with enjoyment and negative relations with anxiety. Indications for the negative relationship between self-efficacy and anxiety have also been found in school subjects other than PE and in the context of athletic competitions [90,91,92,93]. The positive relationship between self-efficacy and perceived enjoyment in university courses and PA has been found in samples of university students [49,94]. The negative relation between value appraisals and anxiety has also been identified in a sample of fifth graders in the context of mathematics [95]. Empirical support for the assumed positive relationship between intrinsic value and enjoyment has been provided in a sample of university students [49]. The high regression weight of enjoyment on intrinsic value (Figure 2) and the strong latent correlation (Table 1) between the two constructs could raise some doubts regarding their unique contributions to the proposed model of the present study. Based on similar concerns, Simonton and Garn [49] addressed the conceptual similarities of intrinsic value and enjoyment. Several studies have measured intrinsic value based on the expectancy-value theory of motivation [49,96], in which intrinsic value is characterized as students’ enjoyment of a task or domain [96]. Researchers using intrinsic value items based on the expectancy-value theory of motivation therefore tend to use terminology and address contents that are also found in enjoyment items. Consequently, the respective manifest items measure highly similar latent constructs in these cases, which would explain the conceptual overlap between intrinsic value and enjoyment [49]. Therefore, to prevent a potential overlap between these two constructs, Pekrun [36] makes a clear distinction between intrinsic value and enjoyment by characterizing intrinsic value as an antecedent of achievement emotions. Like in the study by Simonton and Garn [49], the scales used in the present study adhered to this distinction. In both studies, this approach resulted in highly correlated but distinct constructs since intrinsic value and enjoyment exhibited discriminant validity.

With regard to a potentially PA-enhancing composition of achievement emotions, it is important to note that the cognitive appraisals added to each other in a complementary way, since self-efficacy was particularly important for the reduction of anxiety in PE whereas the intrinsic value ascribed to PE was a major positive predictor of the enjoyment the students experienced in class. This shows that when students feel more confident to perform an action, they experience less anxiety. Furthermore, students’ interest in the activity seems to be of high importance for enjoyment. These results are in line with CVT-based assumptions, showing that positive appraisals of self-efficacy and intrinsic value can evoke positive achievement emotions and are able to reduce negative achievement emotions [36].

In line with Hypothesis 3, both of the assessed achievement emotions were significant predictors of leisure-time PA. However, the role of enjoyment as a positive predictor was by far more important than the role of anxiety as a negative predictor. Both results align with previous findings. Whereas enjoyment was consistently identified as a powerful trigger for PA [56,59,97,98,99,100], ambiguous findings were shown for anxiety [61,62,92]. In the present study, almost one quarter of the variance in leisure-time PA was explained by enjoyment in PE. This is even more remarkable in view of the fact that the predicting variable exclusively refers to processes and experiences in the context of PE, but still managed to explain a substantial amount of variance in a behavior taking place outside of school. The fact that, although being a statistically significant predictor, anxiety in PE only explains two percent of the variance in leisure-time PA suggests that affective experiences made in PE provide more chances than risks with regard to their effects on PA during leisure time. Therefore, PE can be seen as a potentially powerful platform for the promotion of leisure-time PA, especially if it is conducted in a way that evokes regular positive achievement emotions in students while keeping negative ones on a minor level.

As proposed by Hypothesis 4, the significant indirect effect of teacher autonomy support on leisure-time PA via cognitive appraisals and achievement emotions (Table 2) provides an example for how PA-enhancing achievement emotions can be triggered in the context of PE. However, it is necessary to apply several teaching strategies in order to provoke further PA-enhancing chains of effects, since students’ enjoyment and the overall level of positive experiences in PE decrease with age [100,101,102].

### 4.2. Strengths and Limitations

Based on the theoretical framework of the CVT [36], this study aimed to provide new insights in lower-track secondary school students’ emotional experiences in PE. Additionally, the antecedents of emotions were examined with regard to both students’ learning environment and their subjective control-value appraisals. The applied holistic approach was completed by examining the consequences of students’ emotional experiences with respect to their PA behavior in leisure time. To the authors’ knowledge, this holistic approach had not been applied in the context of PE before. A further strength is the examination of the proposed model in the specific population of lower-track students mainly stemming from households with a SES below average. Autonomy support as a representation of the learning environment was measured in multidimensional manner [68,69], thus providing insights into the effectiveness of different teaching strategies. In contrast to the majority of previous studies, value appraisals were measured separately, which has been recommended to enable a focus on the unique role of students’ appraisals of intrinsic value as an antecedent of achievement emotions experienced in PE [62,95]. Finally, given the substantial concern about the role of PE as a facilitator of students’ leisure-time PA [12,13], this study suggests a substantial potential of emotional experiences in PE as a powerful predictor of PA behavior outside of school.

Some limitations yet should be considered. The cross-sectional design does not allow for definite conclusions regarding causal effects. Future research could adapt a residual change score approach to measure change in constructs over time while controlling for their covariance stability [103]. Leisure-time PA was measured by students’ self-reports. The use of accelerometer-based measurements might have provided a deeper understanding of students’ PA levels and patterns in leisure time [104]. Although evidence for the validity of the scale used to assess autonomy support was provided [68], it cannot be guaranteed that every participant in fact rated perceived autonomy support. Using self-report instruments does not necessarily capture the actual teacher behavior but reflects a participant’s internal representation that is triggered by the content of the items. This representation can be affected by different conditions [105]. Although autonomy support can be rated with regard to concrete teaching behaviors [69,87,106] that are learnable [107] and despite the detailed introduction that participants received before completing the questionnaire, it is possible that some students rather expressed their general approval or disapproval of the teacher or the lesson as being good or bad from a more emotional and less specific standpoint. Approval of the teacher or the lesson may, for example, depend on teachers’ personality traits, such as extraversion, openness or rather motivational characteristics like enthusiasm. Thus, it may be assumed that in some cases students’ ratings of autonomy support could be biased or even replaced by their ratings of teacher personality. Therefore, it would be informative to add direct or video-based observations of teachers’ autonomy support in PE to the perceived autonomy support reported by the students [87,108]. Furthermore, it would be interesting to integrate other CVT-based achievement emotions into the proposed model since each emotion may be determined by different types of appraisals and have different consequences [36]. Provided that the questionnaire would not exceed a reasonable length for the respective study sample, also including extrinsic value in the assumed model would extend the understanding of achievement emotions [49]. Generally, this study assessed students’ trait emotions, which are more general and relate to interpersonal differences in the experience of emotions. A state emotion on the other hand is closer to the emotional experience [109]. Although in academic situations measuring trait emotions may indeed be more useful to describe and explain their impact on learning and outcome [109], one-time trait surveys can be influenced by subjective beliefs, since the participant has to rely more on semantic rather than on episodic knowledge, which eventually allows limited conclusions about students’ current state of emotions [105]. To allow a direct self-report in the respective situation, future studies could include diary studies [110] or experience time sampling [111] in their assessments. Furthermore, even if self-reports are regarded as standard tools for measuring emotions in school settings, it would be interesting to combine behavioral and neurophysiological assessment tools with video-based PE lesson studies to capture all components of emotions [109,112].

## 5. Conclusions

The importance of high-quality PE in schools is well known. Positive emotional experiences in PE could be seen as a main factor to increase PA in a lifelong perspective and could thus help students to improve their overall health. The findings of this study indicate that PE teachers have the opportunity to create positive emotional experiences for students and to reduce the experience of negative emotions by use of autonomy-supportive teaching strategies. It is further shown that behavior of the PE teacher does not directly lead to positive or negative student achievement emotions. Instead, teacher autonomy support first affects the students’ appraisals of control and value. If these appraisals tend to be positive, the possibility of experiencing positive and activating achievement emotions is increased. The results suggest that PE exhibits the potential to affect students’ thoughts and feelings related to PA in leisure time and thus is a promising starting point for children and adolescents with regard to an active lifestyle in the long term.

## Figures and Tables

**Table 1:** Correlations between latent variables and composite reliability coefficients.

Variable	1.	2.	3.	4.	5.	6.	7.	8.
1. Cognitive autonomy support	**0.87**							
2. Procedural autonomy support	0.67 **	**0.81**						
3. Organizational autonomy support	0.61 **	0.55 **	**0.84**					
4. Academic self-efficacy	0.32 **	0.23 **	0.25 **	**0.85**				
5. Intrinsic value	0.35 **	0.24 **	0.20 **	0.61 **	**0.93**			
6. Joy	0.47 **	0.35 **	0.28 **	0.55 **	0.79 **	**0.95**		
7. Anxiety	−0.16 **	−0.14 **	−0.10 *	−0.45 **	−0.37 **	−0.42 **	**0.84**	
8. Physical activity	0.20 **	0.11 **	0.15 **	0.52 **	0.64 **	0.49 **	−0.24 **	**0.95**

Note. Composite reliability coefficients for each variable are shown in bold on the diagonal. * = *p* < 0.01, ** = *p* < 0.001.

**Table 2:** Standardized parameter estimates for the indirect effects from the partial least squares analysis.

Independent variable	Dependent variable	Mediator(s)	β	*p*	ES
Cognitive autonomy support	Enjoyment	Academic self-efficacy	0.27	<0.001	0.13
		Intrinsic value			
Cognitive autonomy support	Anxiety	Academic self-efficacy	−0.15	<0.001	0.02
		Intrinsic value			
Cognitive autonomy support	Physical activity	Academic self-efficacy	0.14	<0.001	0.03
		Intrinsic value			
		Enjoyment			
		Anxiety			
Academic self-efficacy	Physical activity	Enjoyment	0.08	0.006	0.04
		Anxiety			
Intrinsic value	Physical activity	Enjoyment	0.34	<0.001	0.22
		Anxiety			

Note. ES = Effect size estimate.

## Data Availability

The data presented in this study are available as Supplementary File S1.

## Supplementary Materials

The dataset analyzed for this study can be found as supplementary file S1 online at https://www.mdpi.com/article/10.3390/ijerph18083987/s1.
